# Silent but Severe: A Case Report of a Large Retroperitoneal Hematoma Following Routine Intradetrusor Botulinum Toxin A Injections

**DOI:** 10.7759/cureus.90013

**Published:** 2025-08-13

**Authors:** Juho T Eineluoto, Jutta Jokinen

**Affiliations:** 1 Department of Urology, Hyvinkää Hospital, Hyvinkää, FIN; 2 Department of Urology, University of Helsinki and Helsinki University Hospital, Helsinki, FIN

**Keywords:** botulinum toxin, botulinum toxin a, cystoscopy, multiple sclerosis, neuro-urology, pelvic hematoma

## Abstract

Intravesical botulinum toxin A injections are an established and generally well-tolerated treatment for an overactive bladder. Major complications are uncommon. We report a rare case of a substantial retroperitoneal hematoma following routine intradetrusor botulinum toxin administration. A 53-year-old female with a diagnosis of multiple sclerosis was treated at the urology outpatient clinic for neurogenic bladder symptoms. Four days after the injections, she presented to the emergency department with lower abdomen pain, hematuria, urinary retention, and a visible hematoma around the umbilicus (Cullen’s sign). Her hemoglobin level had decreased from 123 g/L to 107 g/L. A CT scan revealed a large pelvic retroperitoneal hematoma measuring 11 × 5 × 5 cm, attributed to the injections. She was managed conservatively. A follow-up CT scan two months later demonstrated a reduction in the size of the hematoma.

## Introduction

Flexible office cystoscopy (FC) performed under local anesthesia has been commonly used among urologists since 1973, when the first flexible prototype was introduced [1]. FC can be used for various indications, including visual assessment of the bladder and urethra, biopsy and electrocoagulation of mucosal regions of interest, and overactive bladder (OAB) treatment [2,3]. Intradetrusor botulinum toxin A (iBotox) injections were first administered during cystoscopy in 2000 among patients with spinal cord injury and neurogenic OAB [4]. This effective method has rapidly spread throughout the world. The FDA has approved iBotox injections for the treatment of neurogenic OAB and idiopathic detrusor overactivity, especially in conditions unresponsive to lifestyle changes and anticholinergic medicine [2,5-7].

The complications of iBotox injections are often minor and seldom require medical attention. They include urinary retention (0-33%), occasionally leading to intermittent self-catheterization (6-88%), mild hematuria (2-21%), urinary tract infections (14%), and pain at the injection site [2,8-10]. Systemic side effects are extremely rare, but botulinum toxin A can spread to distant places and cause generalized weakness, blurred vision, and diplopia [2,11].

Atayi et al. recently published the first documented case of a rare complication following iBotox injections, involving the development of a large pelvic hematoma that required clinical evaluation and serial CT imaging for follow-up [12]. Given the widespread use of iBotox therapy over several decades, it is possible that this potentially serious complication has been underrecognized and underreported. In this article, we present a case similar to that described by Atayi et al., further illustrating this uncommon complication.

## Case presentation

The patient was a 53-year-old female who was diagnosed with multiple sclerosis in her 30s. At an early stage of the disease, she presented with OAB symptoms. Due to insufficient symptom control, a combination therapy with anticholinergic fesoterodine 4 mg once daily and sympathomimetic mirabegron 50 mg once daily was soon introduced, but this regimen failed to provide adequate relief. Subsequently, iBotox therapy was implemented. The initial dose consisted of 100 units of Allergan Botox®. This treatment resulted in improved control of OAB symptoms. However, the therapeutic effect lasted only four to five months. Over time, the patient’s iBotox dose was gradually increased due to the diminishing duration of efficacy. She has now been receiving iBotox injections for neurogenic OAB for more than five years. Her current regimen consists of 300 units of Allergan Botox®, diluted in 10 mL of saline and injected into 20 sites within the bladder wall, administered at six-month intervals.

She was not on antiplatelet or anticoagulation medicine. A preoperative urine sample revealed no bacterial growth. The medicine was administered via local anesthetic FC using a multi-use cystoscope. The procedure was performed by a specialist urologist with more than seven years of expertise in intradetrusor injections. A 70 cm long 23 gauge cystoscopy needle (injeTAK®, Laborie Medical Technologies, Portsmouth, NH) with an adjustable 4.8 French needle tip was set to 4 mm, and 20 injections were given around the detrusor muscle, avoiding the area of the urethral openings, trigone, and bladder dome. No perioperative complications were detected.

Twelve hours later, the patient contacted the emergency room (ER) over the phone and asked for advice. She complained of pain in the lower abdomen, shivering, and hematuria. Previously, no such symptoms were detected after treatment. The patient was asked to visit the ER for further examination. One day later, at the ER, she met a nurse who measured her residual urine to be 230 mL. Her stomach pain was still tolerable, so she was advised to start intermittent self-catheterization, which she had been taught previously. She was discharged later the same day.

**Figure 1:**
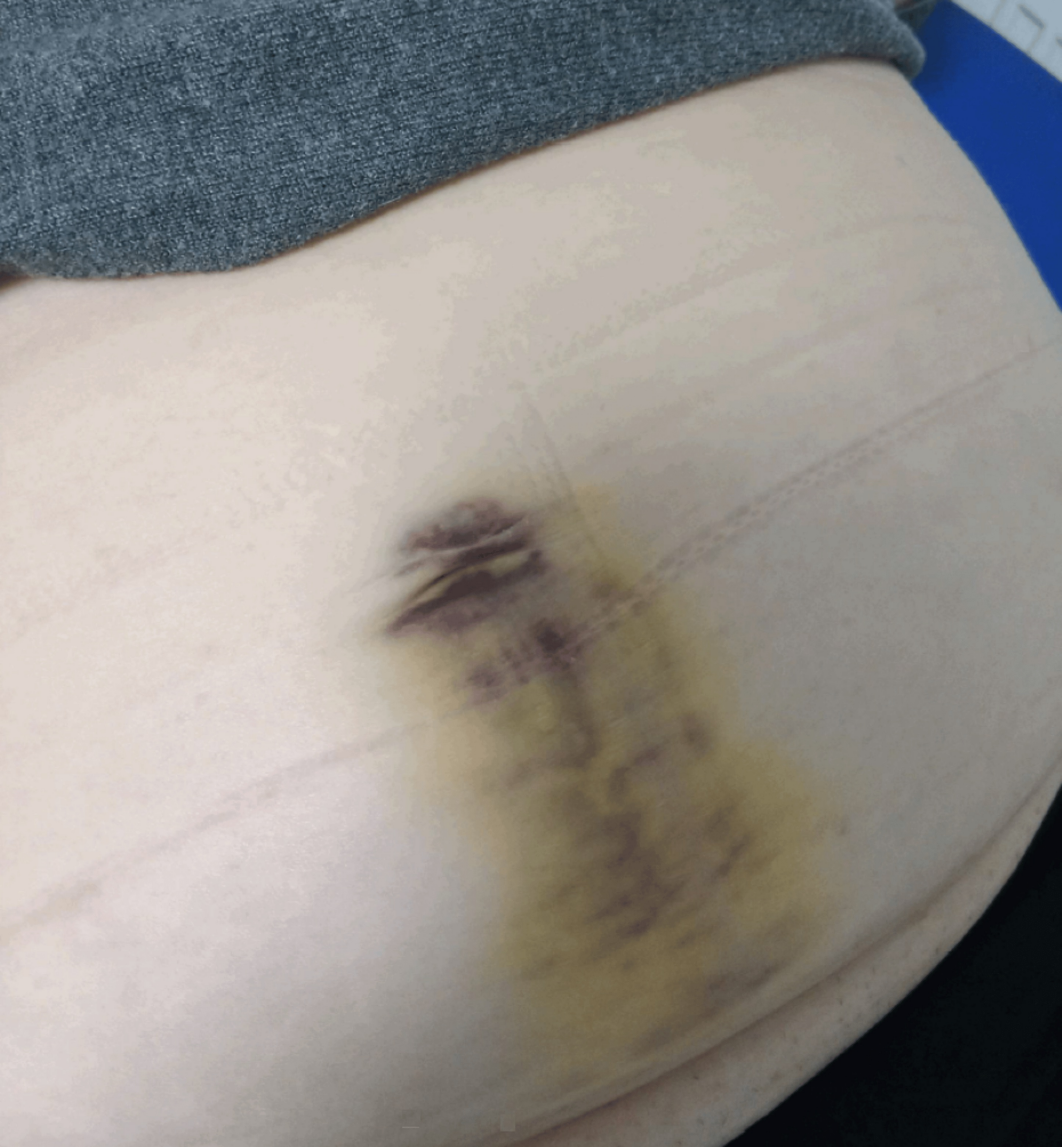
A visible hematoma was observed in the periumbilical and suprapubic regions (Cullen’s sign) four days after the intradetrusor botulinum toxin A injections. The patient presented with periumbilical pain and difficulty voiding.

**Figure 2:**
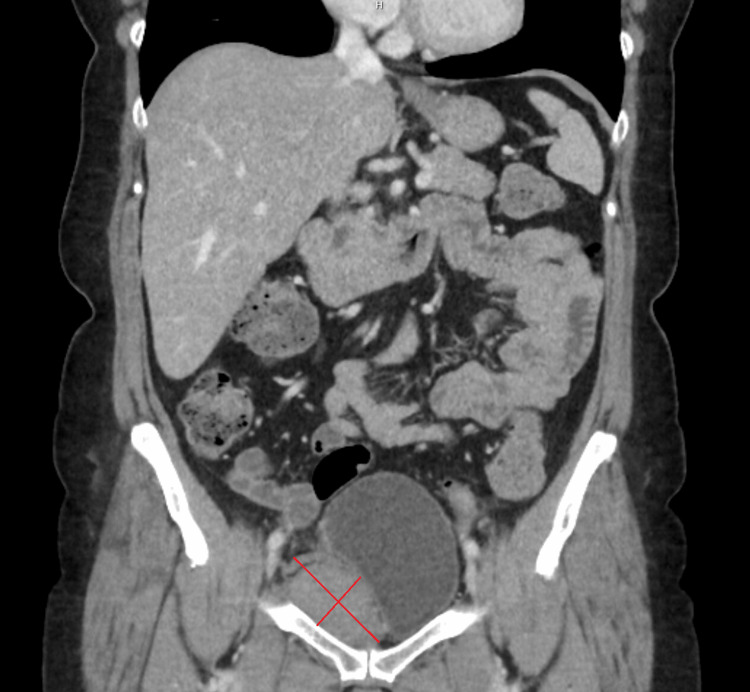
A computed tomography performed four days after intradetrusor botulinum toxin A injections revealed a large pelvic hematoma dislocating the bladder. Urethral dislocation, in combination with the intradetrusor injections, resulted in the need for intermittent catheterization.

**Figure 3:**
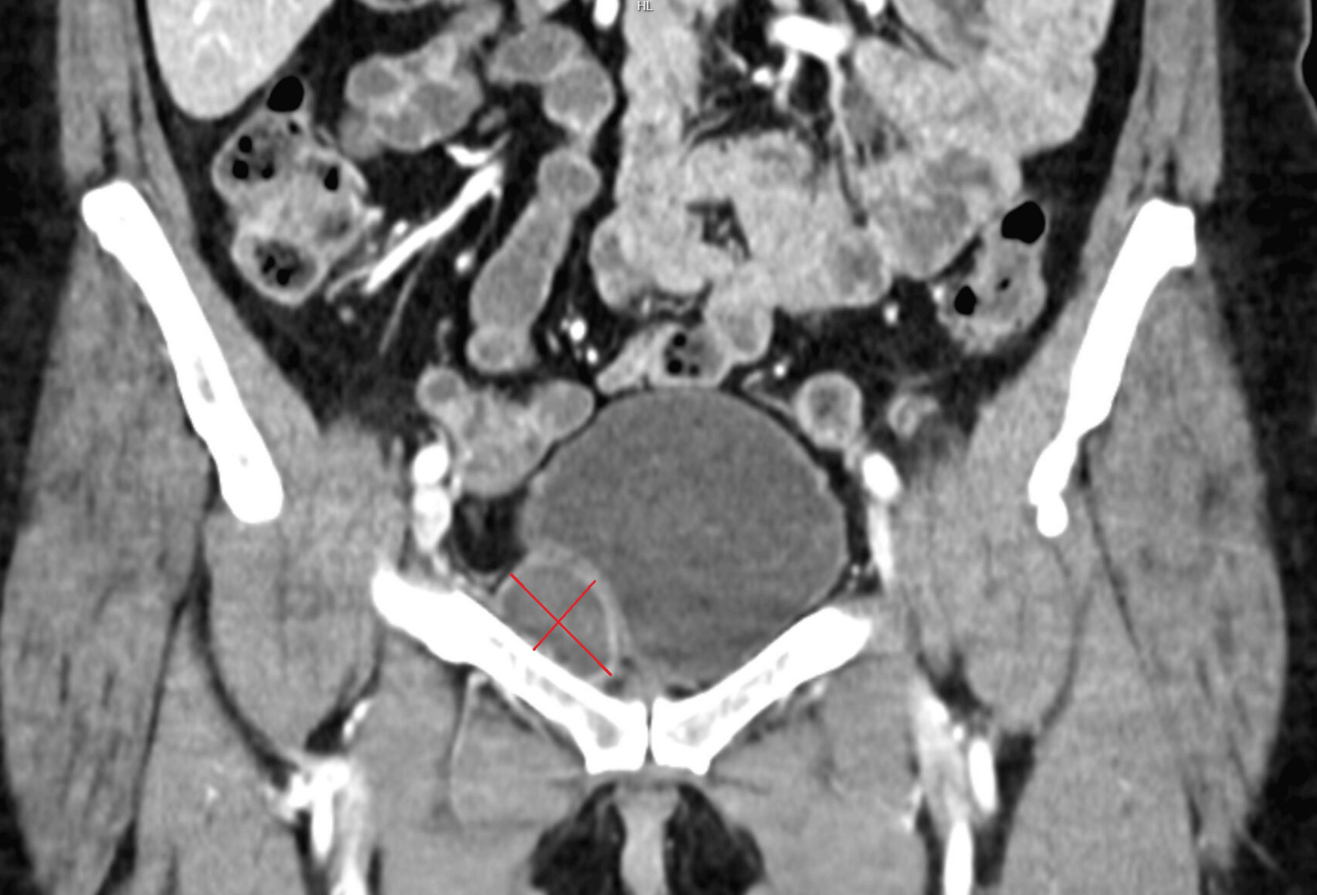
A follow-up CT scan two months after the initial treatment revealed a smaller, organized, hypodense hematoma. At that time, the patient did not report any pain.

Four days after the procedure, she presented at the ER with increased abdominal pain. She experienced pain from the torso movement and turning in bed. She met a doctor this time, and a number of laboratory exams were taken. Her laboratory values demonstrated a decrease in hemoglobin and hematocrit counts (Table 1). There was no record of coagulation laboratory testing performed within the past five years. The most recent prior hemoglobin measurement, taken two years earlier, indicated a decline of 16 g/L. A visible hematoma was observed around her umbilicus and lower abdomen (Cullen’s sign) (Figure 1), and she experienced tenderness when this area was palpated. An abdominal CT scan with arterial and venous contrast phases revealed a perivesical pelvic retroperitoneal hematoma (11 × 5 × 5 cm) dislocating the bladder on the right side (Figure 2). Vascular abnormalities around the bladder, such as aneurysms, were not visible in the CT scan. The urine sample showed unspecified *Staphylococcus* bacterial growth. As her condition was stable, she was discharged the same day, with a recommendation to take 1000 mg of paracetamol and 800 mg of ibuprofen one to three times daily when needed. A cephalexin 500 mg antibiotic tablet three times daily was prescribed, and an ultrasound of the lower abdomen was ordered as a follow-up. Eight days later, an ultrasound was performed, and the hematoma had shrunk (10 × 3.8 cm). She was contacted via phone, and as her symptoms slowly diminished, she was advised to discontinue antibiotic therapy that day. An abdominal CT scan was ordered two months after the procedure, and a smaller (8 × 2.7 cm), organized, hypodense hematoma was observed (Figure 3).

**Table 1:** Laboratory results before and after intravesical intradetrusor toxin A injections. * Abnormal result.

	Four days after injections	Before injections	Normal range
Hemoglobin	107*	132	117–155 g/L
Hematocrit	32*	38	35–46%
Leucocytes	7.9	4.7	3.4–8.2 E9/L
Platelets	301	231	150–360 E9/L
Creatinine	58	74	50–90 µmol/L
Urine sample	Unspecified *Staphylococcus* bacteria*	No bacterial growth	No bacterial growth
C-reactive protein	6	Not available	<4 mg/L

## Discussion

We report the largest published retroperitoneal hematoma to date arising from iBotox injections administered via outpatient FC.

A slightly smaller pelvic hematoma after iBotox injections was first reported by Atayi et al. [12]. Consistent with their case, we also used 20 bladder injections, and our needle length was set to 4 mm. The symptoms after the injections were similar between these two patients, consisting mainly of lower abdominal pain and a visible hematoma around the umbilicus and suprapubic area. In contrast to their case, our patient presented with visible hematuria and significant urinary retention necessitating intermittent self-catheterization. Additionally, the urine sample in our case showed bacterial growth requiring antibiotic treatment. While Atayi et al. reported their dose as 100 units of Allergan Botox®, our patient received her standard 300 units of the same compound. This may explain the observed urinary retention, although our hematoma dislocated the bladder, possibly hampering urine outflow. However, our patient had been receiving a similar treatment of 20 injections of 300 units of Allergan Botox® for four years with no complications and no need for intermittent self-catheterization. Atayi et al. reported a decrease of hemoglobin of 37 units, whereas we observed a decrease of 16 units. Neither of these patients has thus far required operative treatment due to this complication.

The typical bladder wall thickness among women is approximately 2-3 mm [12,13]. Upon distension, the bladder wall layers compress, potentially reducing the overall thickness to less than this range. We hypothesize that the iBotox needle, set to 4 mm depth at the time of administration, penetrated the full thickness of the bladder wall, resulting in a puncture of a vessel in close proximity external to the bladder wall. While multiple vascular sources could account for the bleeding, we estimate that the injured vessel was likely a branch of the inferior vesical artery, the vaginal artery, or the vesical venous plexus. Therefore, reducing both the needle depth to 3 mm and limiting the number of injections to 10 instead of 20 may reduce the likelihood of hematoma development. Cephalexin 500 mg three times daily was initiated after the patient presented to the ER with bacterial growth identified in a urine culture. To the best of our knowledge, there is currently no scientific evidence supporting the use of antibiotic therapy in cases of suspected infection following iBotox administration and subsequent hematoma formation. Therefore, this treatment was initiated as empiric prophylaxis based primarily on expert opinion rather than evidence-based guidelines. Further studies are needed to establish appropriate management strategies in such cases.

Today, there are over one million cystoscopy procedures performed in the USA alone, and FC has become the cornerstone of urological diagnostics [14]. Severe complications after iBotox injections in outpatient FC settings are rare, with a mortality rate of approximately 0% [9]. In addition to the present case, only a single published report has documented a comparable unusual complication [12]. This implies that this complication may be more frequent than previously thought. The balance between relieving OAB symptoms and avoiding complications at the same time is a challenge. For adequate symptom relief, botulinum toxin A should be distributed around the bladder evenly, with as few injections as possible. It is unclear whether 10 injections are less painful than 20 injections [15]. The risk for vessel puncture increases with more injections.

## Conclusions

OAB symptoms have been treated for decades with iBotox, a therapy that is both effective and generally well-tolerated by patients. Side effects of the injections are typically transient and confined to the bladder. Patients with OAB typically receive injections at three to 12-month intervals, multiple times over their lifetime, underscoring the need for safe and precise application of botulinum toxin in the bladder. A large pelvic hematoma following iBotox injections is a rare but potentially severe complication that requires prompt medical evaluation. Repeated visits to the emergency department, diagnostic imaging, and follow-up appointments can be distressing for patients and increase the economic burden on the healthcare system.

To reduce the risk of retroperitoneal hematoma formation, we recommend using a 3 mm needle and limiting the number of injections to a maximum of 10, particularly in elderly and frail patients. We have adopted this approach in our daily clinical practice and have observed equally effective treatment responses, with no further cases of pelvic hematoma.
